# Stand age influences the climate response and growth resilience of *Picea wilsonii* to extreme drought and cold events

**DOI:** 10.3389/fpls.2026.1884608

**Published:** 2026-07-07

**Authors:** Hanxue Liang, Yayuan Che, Yimin Ren, Jinji Li, Le Wang, Xinlei He, Libo Sun, Zhitao Wu

**Affiliations:** 1 Institute of Loess Plateau, Shanxi University, Taiyuan, China; 2 College of Archeology and Museology, Shanxi University, Taiyuan, Shanxi, China

**Keywords:** dendrochronology, extreme climate events, growth resilience, Loess plateau, radial growth

## Abstract

Global climate change has intensified the frequency and severity of extreme climate events, posing significant threats to forest ecosystem stability. While the impacts of drought have been extensively studied, the comparative resilience of trees to different types of stressors (such as extreme cold) and how these responses vary across developmental stages remains poorly understood. In this study, two standard chronologies of middle-aged and mature *Picea wilsonii* in the eastern Loess Plateau were developed and climate-growth relationships were assessed via bootstrapped correlation, and calculated resistance, recovery, and resilience indices were compared using one-way ANOVA. Results showed that middle-aged growth is primarily promoted by moisture during the late growing season (July–September), whereas mature trees are governed by pre-growing season moisture levels. Under extreme drought, middle-aged stands exhibited higher resistance but lower recovery than mature stands; this pattern was reversed under extreme cold, with both age classes reaching comparable resilience. Intra-stand analyses revealed that middle-aged trees have lower resistance but higher recovery and resilience under cold than drought. Conversely, mature trees showed higher resistance and resilience under cold than drought, but similar recovery across stressors. These findings indicate that different age classes follow divergent stabilization strategies: an opportunistic strategy in middle-aged trees versus a conservative strategy in mature trees. These findings indicated that management should be age-specific, prioritizing drought mitigation for younger stands and protecting the structural integrity of mature forests to ensure their function as stable carbon reservoirs in a changing climate.

## 1 Introduction

In recent decades, global climate warming has intensified, leading to a significant increase in the frequency, severity, and duration of extreme climate events (Jing et al., 2026; Li et al., 2024). These perturbations, such as mega-drought as well as cold-hot fluctuations, have exerted profound impacts across various dimensions of terrestrial ecosystems (Gao C. et al., 2025; Lu et al., 2025). Forest ecosystems, which play a critical role in global carbon sequestration and climate regulation, are particularly vulnerable to these climatic anomalies (Jing et al., 2026; Zhu et al., 2023). Consequently, widespread forest decline, growth reductions, and large-scale mortality episodes have been increasingly reported on a global scale, ranging from semi-arid biomes to humid temperate and tropical regions (Belokopytova et al., 2022; Rubio-Cuadrado et al., 2025; Serra-Maluquer et al., 2018). Such deterioration not only impairs the provisioning of essential ecosystem services but also threatens to shift forests from carbon sinks to carbon sources, potentially creating a positive feedback loop that further accelerates climate change (Wang et al., 2022; Zhu et al., 2023).

Given these escalating threats, quantifying the ecological stability of forests—specifically their resilience to extreme disturbances—has become a paramount research priority (Liu et al., 2023; Piraino et al., 2022; Wang et al., 2022). Growth resilience is broadly defined as the capacity of an organism or ecosystem to withstand perturbations and restore its pre-disturbance structure and function (Lloret et al., 2011). Since the landmark proposal by Lloret et al. (2011) of a unified quantitative framework based on tree-ring data, forest stability has been widely assessed using three primary indices: resistance (*R_t_*), the ability to maintain growth during a disturbance; recovery (*R_c_*), the capacity to increase growth after the event; and resilience (*R_s_*), the ability to reach pre-disturbance growth levels. Following the introduction of these metrics, extensive research has explored the drivers of forest resilience across diverse scales, from individual tree traits to global biomes (Bouknine et al., 2026; DeSoto et al., 2020; Duan et al., 2022; Fang and Zhang, 2024; Jiang et al., 2025). Studies have demonstrated that resilience is modulated by a complex interplay of extrinsic factors, such as drought intensity and local topography (Han et al., 2025; Liang et al., 2024), and intrinsic factors, including species-specific physiological strategies (Belokopytova et al., 2022; Duan et al., 2022), tree size (Ding et al., 2017; Serra-Maluquer et al., 2018), forest structure (Italiano et al., 2024), inter-specific facilitation (Pretzsch et al., 2013), and stand age (Popa et al., 2024; Wang et al., 2022).

While much of the existing literature has focused on extreme drought, emerging studies have begun to contrast these responses with other stressors, such as late spring frosts, revealing distinct trade-offs between growth resistance and recovery across different species and developmental stages (Príncipe et al., 2017; Vitasse et al., 2019; Zhu et al., 2023). Understanding these differentiated response patterns is essential for predicting future forest dynamics and developing adaptive management strategies to enhance forest stability in an increasingly uncertain climate.

The Loess Plateau is one of the regions most sensitive to climate change. Given its vast expanse and significant variations in precipitation and temperature across different areas, the climatic responses of forests and their growth resilience to extreme climate events also vary by region. Consequently, large-scale research is necessary to comprehensively assess the impact of climate change on the forests of the Loess Plateau. In recent years, extensive dendrochronological research has been conducted on various key tree species across the Loess Plateau, including *Picea crassifolia* (Gao C. et al., 2025), *Pinus tabulaeformis* (Cai et al., 2014), *Robinia pseudoacacia* (Yan et al., 2025), *Larix principis-rupprechtii* (Guan et al., 2007), etc. These studies have revealed that species-specific physiological traits and geographical heterogeneity significantly modulate growth-climate relationships (Liang et al., 2026). Among the various environmental drivers, temperature, precipitation, and drought indices such as the Standardized Precipitation Evapotranspiration Index and Palmer Drought Severity Index are the most frequently reported critical factors limiting radial growth in this region (Gao C. et al., 2025; Liang et al., 2026).

Although a substantial amount of research has been conducted, studies have been relatively concentrated in the arid northwestern regions (Gao W. et al., 2025; Gou et al., 2023; Liang et al., 2021; Liu et al., 2017; Wang et al., 2022), while research on the eastern semi-arid to semi-humid regions remains relatively scarce (Han et al., 2025; Chen et al., 2023; Yan et al., 2025). Furthermore, while research on responses to climate change is abundant (Li et al., 2026; Sun et al., 2021; Xie et al., 2024), studies on growth resilience to extreme climate events remain scarce (Han et al., 2025; Jing et al., 2026).

Pangquangou Mountain, located in the eastern Loess Plateau, is an important nature reserve and biodiversity hotspot. Forests play a crucial role in soil and water conservation and the maintenance of biodiversity. Studies on the response of local forests to climatic factors have been conducted, and it has been found that spring precipitation may be a key limiting factor for tree growth (Huang et al., 2021), and ENSO may be the main forcing (Huang et al., 2024). However, it is still insufficient: it remains unclear how stands of different ages respond to climatic factors, and the growth resilience of different forest age classes to extreme drought and extreme cold events is even less understood. Therefore, in the Pangquangou Mountain area, we selected *Picea wilsonii* as our study subject. *P. wilsonii* is a key community-forming species widely distributed in the high-altitude zone (1900–2600 m) of the Pangquangou Mountain area. As a dominant conifer species in the cold-temperate subalpine forest, it is characterized by strong shade tolerance, high cold resistance, and remarkable adaptability, making it an ideal subject for investigating forest responses to extreme climatic events. Currently, studies on this species in the sounding region have mainly focused on growth-climate responses (Huang et al., 2024; Liang et al., 2026), while growth resilience to extreme climatic events has not been reported. Thus, we collected samples from two stands of different ages to investigate their responses to climatic factors using dendrochronology methods. Concurrently, we examine differences in the growth resilience of the two stands to various extreme drought and cold events. We hypothesize that the response characteristics of the two age classes to climate and extreme climate events will differ significantly.

## 2 Materials and methods

### 2.1 Study area

Pangquangou Mountain (111°22′–111°33′ E, 37°45′–37°55′ N) is located in the central section of the Luliang Mountains in western Shanxi Province, in the eastern part of the Loess Plateau, at an elevation of over 1,500 meters. The climate belongs to the warm temperate semi-humid zone, representing a transitional climate between the temperate and warm temperate zones. The main tree species include *P. wilsonii*, *Larix gmelinii* var. *principis-rupprechtii*, *Quercus mongolica*, and *Betula platyphylla*, etc. Larix and Picea are the dominant constructive species in high-altitude areas. The forest is a natural secondary forest, with a very small amount of remnant primary forest. As it is located within the Pangquangou National Nature Reserve, strict enclosure and protection measures have been implemented, and it has not been subjected to human disturbance or silvicultural treatment.

### 2.2 Field sampling and tree ring data

**Figure 1:**
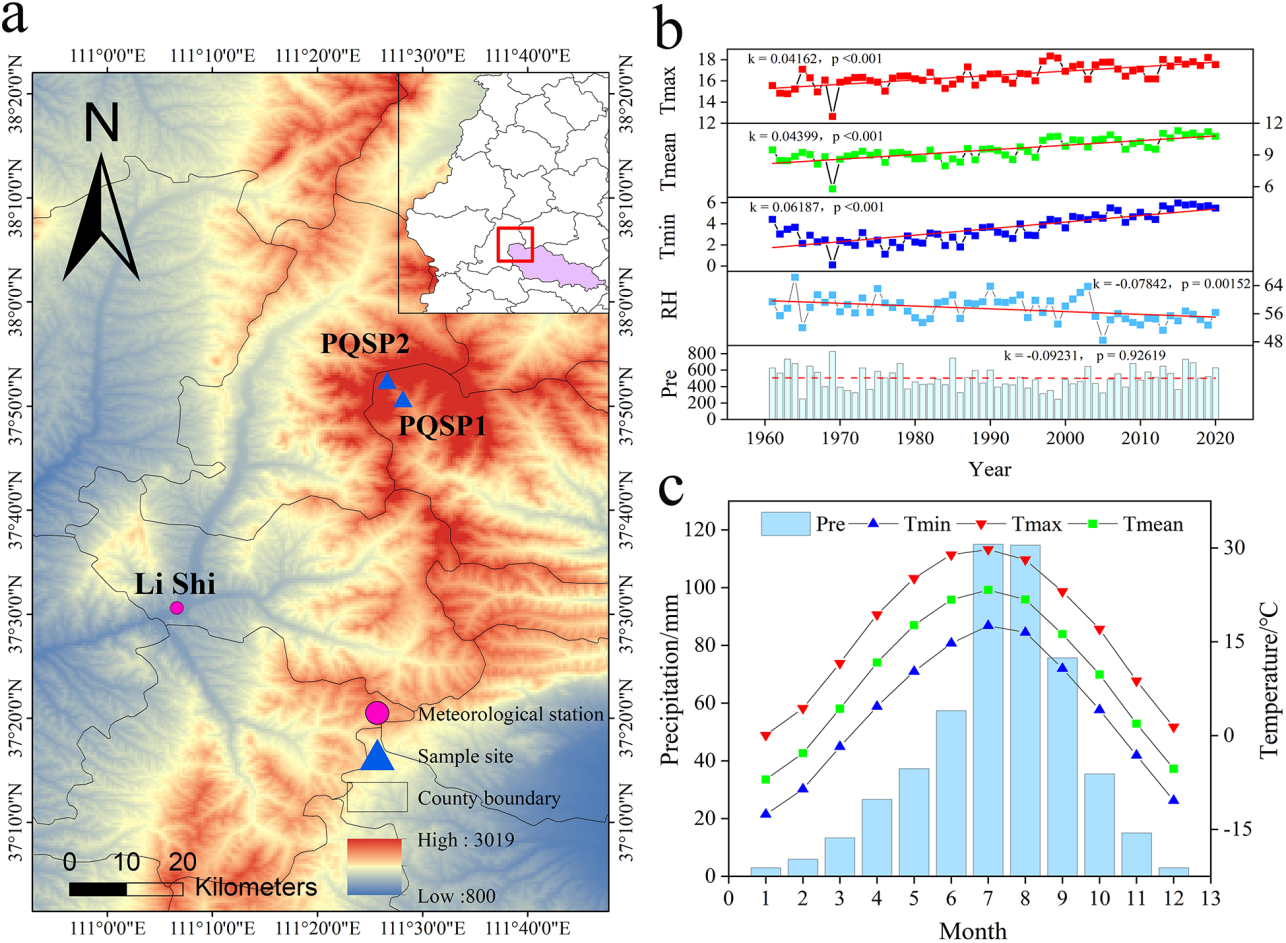
Sampling sites locations and climatic characteristics. **(a)** Location of the sampling sites. **(b)** Annual mean climatic characteristics, including Tmax (monthly mean maximum temperature), Tmean (monthly mean temperature), Tmin (monthly mean minimum temperature), RH (relative humidity), and Pre (monthly precipitation). **(c)** Multi-year mean monthly climatic characteristics.

Undisturbed health *P. wilsonii* trees were selected as sampling subjects. After consultation with the forestry department, two pure forest stands of different ages were selected (PQSP1, 111.468948E, 37.845461N, canopy cover is 74%; PQSP2, 111.443914E, 37.874471N, canopy cover is 91%) (**Figure 1a**). For each stand, we established a 20 m × 20 m sample plot. For each plot, two tree-ring cores from every tree (diameter at breast height greater than 10 cm) were drilled with 5mm diameter increment borers along the vertical slope, with each sample taken as close to the center of the tree as possible. Finally a total of 78 tree cores were collected from 39 trees at PQSP1, while PQSP2 yielded a total of 41 tree cores from 21 trees.

In the laboratory, tree-ring cores were allowed to air-dry naturally before being mounted on wooden blocks using latex. After thorough drying, the samples were sanded with sandpaper of varying grits until the growth rings were clearly visible under a microscope. Images were scanned at 1200 dpi resolution (using MICROTEC 9800XL), and ring widths were measured using CooRecorder 9.0, followed by cross-dating with CDendro 9.0. The dating results were verified using COFECHA software. Then the dplR package in R was used to detrend the tree-ring width sequences from the two stands (using a cubic spline with a 50% frequency–response cutoff equal to 64 years) (Bunn, 2008). Subsequently, a bi-weighted average was calculated to derive the standard tree-ring width chronologies (STD) for the two stands. A subsample signal strength (SSS) exceeding 0.85 was used as the threshold for a stable period. The inter-series correlation coefficient (Rbar), expressed population signal (EPS), and signal-to-noise ratio (SNR) were used as statistical parameters to evaluate chronological quality (**Table 1**).

**Table 1:** Statistical parameters of the two chronologies.

Sites	Trees/cores	Time span	SSS>0.85	Rbar	EPS	SNR
PQSP1	39/78	1965-2021	1970-2021	0.505	0.983	59.26
PQSP2	21/41	1893-2021	1929-2021	0.369	0.953	20.5

For cores that did not reach the pith, the number of missing rings to the pith was estimated using CooRecorder software based on the curvature of the latewood in the innermost ring and the mean width of the innermost five rings, in order to accurately assess tree age.

### 2.3 Climate data and identification of extreme climate events

To investigate the impact of climate on forests, climate data from the nearest Lishi meteorological station were downloaded (from National Meteorological Science Data Center (https://data.cma.cn). Monthly precipitation (mm), relative humidity (%), monthly mean temperature (°C), monthly maximum temperature (°C), and monthly minimum temperature (°C) were selected as climate variables. The meteorological data (1961-2020) show that the monthly average temperature in the study area is 9.44°C, with a minimum of -7.01°C in January and a maximum of 23.3°C in July. Annual precipitation is 509.16 mm, primarily concentrated in July and August. From 1961 to 2020, the annual average temperature, annual average maximum temperature, and annual average minimum temperature all rose significantly, while relative humidity decreased significantly; precipitation, however, showed no significant change (**Figure 1**).

Additionally, Standardized Precipitation Evapotranspiration Index (SPEI) values on 1-month, 3-month, 6-month, 9-month and 12-month scales (SPEI1, SPEI3, SPEI6, SPEI9 and SPEI12) were calculated for use as climate factors. The SPEI values were computed using the “SPEI” package in R (Beguería and Vicente-Serrano, 2017).

Extreme drought events were defined using the 1-month SPEI, with years characterized by an annual mean SPEI ≤ −1.5 classified as extreme drought years.

Extreme low-temperature events were identified using the percentile threshold method. This approach involves statistical ranking of long-term climate data to determine extreme thresholds, offering the advantages of requiring no *a priori* assumption regarding the data distribution type and being adaptable to regional climatic characteristics. If extreme low-temperature years exhibit a continuous distribution pattern (i.e., persisting for more than one year), this entire continuous period is recognized as a single extreme low-temperature event. We selected the period from 1961 to 2020, as recorded by meteorological stations, as the baseline study period. The mean annual minimum temperature anomalies were first computed for each year within this period, and all anomaly values were subsequently ranked. The five years with the lowest ranked mean annual minimum temperature anomalies were ultimately designated as extreme low-temperature years.

### 2.4 Calculation of growth resilience

Since Lloret et al. (2011) proposed the method for calculating growth resilience (Equations 1–3), subsequent studies have made various adjustments. For example, Pretzsch et al. (2013) used undetrended BAI as the input data for growth resilience, whereas Italiano et al. (2024) used detrended BAI. Additionally, DeSoto et al. (2020) compared TRW and BAI. To ensure consistency between climate response and growth resilience, we used tree-ring width indices to calculate growth resilience. For each tree-ring core, resistance (*R_t_*), recovery (*R_c_*), and resilience (*R_s_*) were calculated separately using the following formula (Lloret et al., 2011):

(1) $$\begin{array}{c} R_{t}=E_{x}/PreE_{x} \end{array}$$

(2) $$\begin{array}{c} R_{c}=PostE_{x}/E_{x} \end{array}$$

(3) $$\begin{array}{c} R_{s}=PostE_{x}/PreE_{x} \end{array}$$

In which, $E_{x}$ represents the individual detrended tree-ring width series for the year in which the extreme climate event occurred; $PreE_{x}$ represents the average of the tree-ring width indices for the four years preceding the extreme climate event; and $PostE_{x}$ represents the average of the tree-ring width indices for the four years following the extreme climate event (Anderegg et al., 2015).

### 2.5 Statistical analyses

The relationship between tree growth and climate was assessed by calculating the correlation coefficients between the plot-specific chronologies and selected monthly climate variables based on bootstrapping 1000 times (see 2.3). The months selected ranged from September of the previous year to October of the current year to account for the lag effect of climate influences. One-way ANOVA tests were conducted to evaluate the resilience, recovery capacity, and growth resilience of different forest stands under various types of extreme climate events. All of these analyses were performed using the R software.

## 3 Results

### 3.1 Characteristics of tree ring chronologies

**Figure 2:**
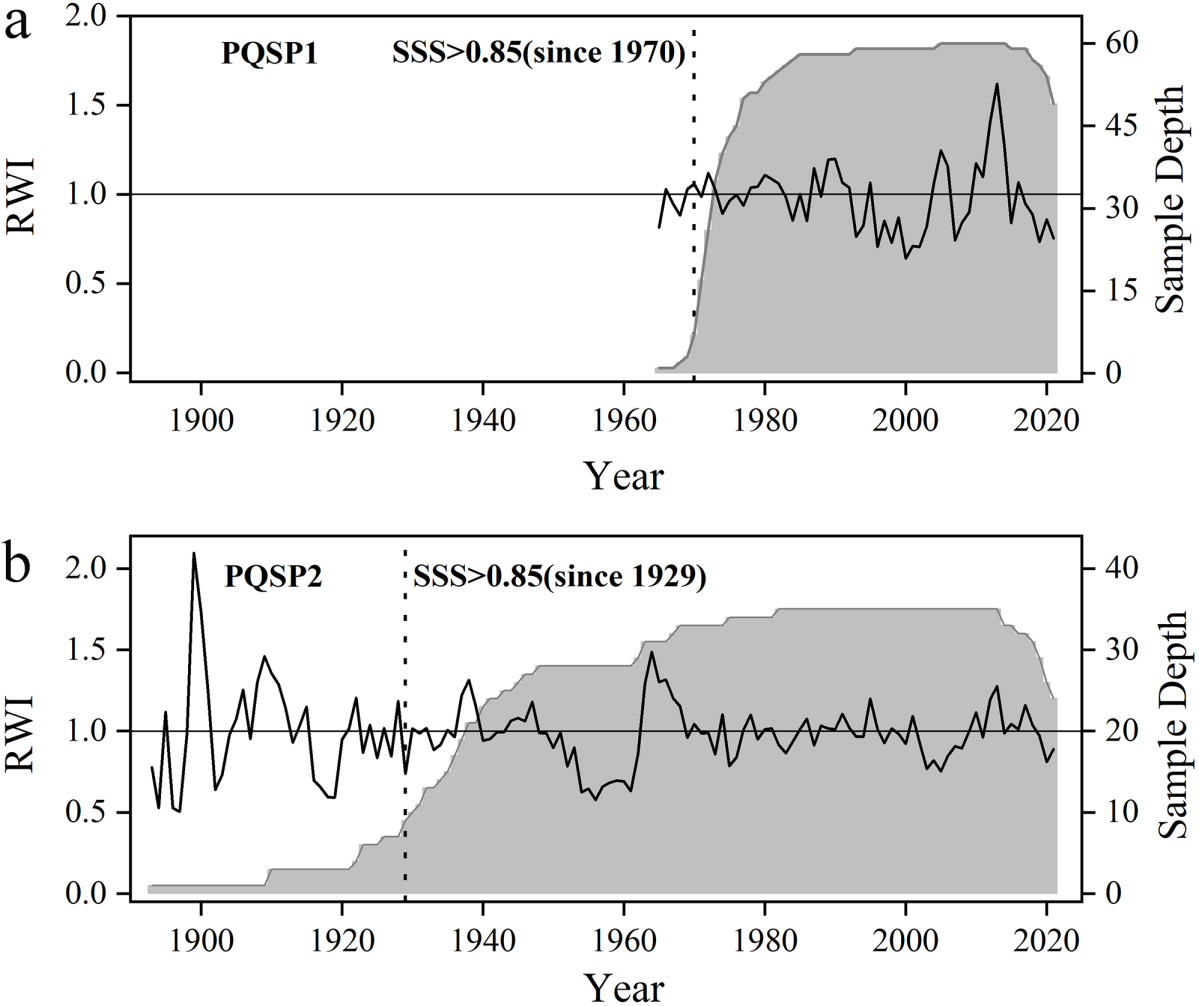
Standard chronologies of the two sampling sites. **(a)** standard chronologies and sampling depth of PQSP1. **(b)** standard chronologies and sampling depth of PQSP2.

The PQSP1 standard chronology spans from 1965 to 2021, with a stable interval of 1970–2021, while the PQSP2 spans from 1893 to 2021, with a stable interval of 1929–2021 (**Figure 2**).

The stand age of PQSP1 was 48.2 ± 5.6 years, and that of PQSP2 was 89.3 ± 18.0 years. There is a significant difference in age class between the two spruce stands. According to the standard, PQSP1 is classified as a middle-aged stand (<60), while PQSP2 is classified as a mature stand (81–120).

Both PQSP1 and PQSP2 have high Rbar values, indicating consistency in the cores. The EPS for PQSP1 and PQSP2 was 0.983 and 0.953, respectively, both exceeding the overall representativeness threshold of 0.85 for tree-ring data. Overall, both chronologies met the criteria for reliability (**Table 1**). The correlation coefficient between the two chronologies was 0.20, indicating no significant correlation.

### 3.2 Identified years of extreme climate events

**Figure 3:**
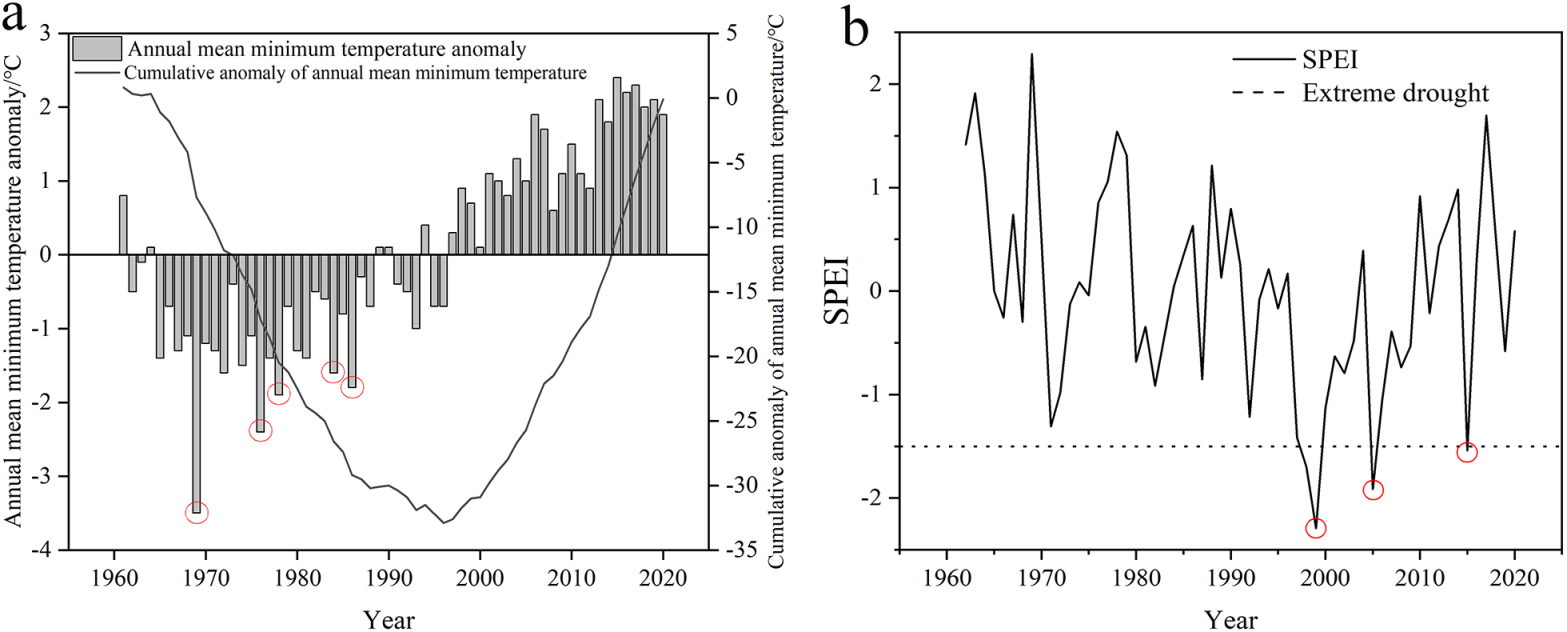
**(a)** Identified years of extreme cold events. **(b)** Identified years of extreme drought events.

Between 1961 and 2020, there were a total of three years of extreme drought, with 1999 being the most severe, followed by 2005, and 2015 being the least severe (**Figure 3**).

Extreme cold events were concentrated in five years: 1969 (3.5°C below normal), 1976 (2.4°C below normal), 1978 (1.9°C below normal), 1984 (1.6°C below normal), and 1986 (1.8°C below normal). However, since the PQSP1 stability chronology begins in 1970, 1969 was not included in subsequent analyses (**Figure 3**).

### 3.3 The response of chronologies to climate factors

**Figure 4:**
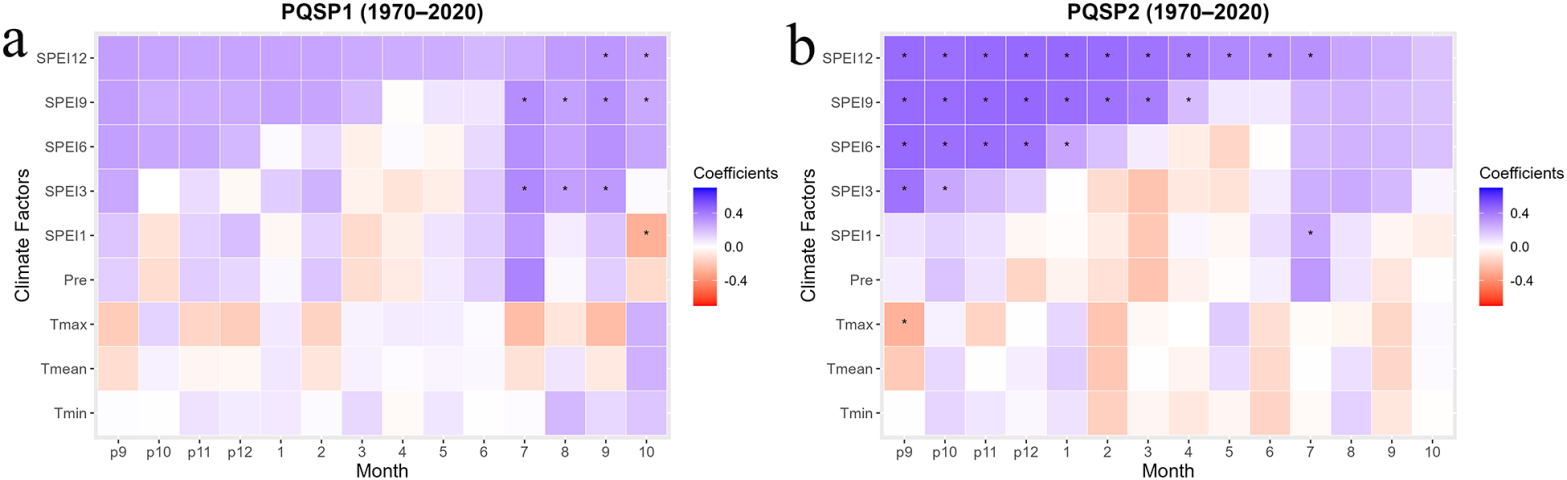
Correlation between chronologies and monthly climate at **(a)** PQSP1 and **(b)** PQSP2 sites. p represents the month of the previous year; SPEI, standardized precipitation evapotranspiration index; Pre, monthly precipitation; Tmean, monthly mean temperature; Tmax, monthly maximum temperature; Tmin, monthly minimum temperature; * indicates a significant correlation at the 0.05 level.

The correlation analysis of the chronologies and climatic factors revealed differences in how the trees in the two stands responded to climatic factors. For PQSP1, the SPEI3, SPEI9, and SPEI12 during the late growing season (from July to September) of the current year significantly promoted tree radial growth. Additionally, the SPEI1 of the current October showed a significant negative correlation with the tree-ring chronology, whereas the SPEI9 and SPEI12 of October exhibited significantly positive effects on tree growth (**Figure 4**).

For PQSP2, the water conditions prior to the growing season were the main factors significantly affecting tree growth. The SPEI3 of previous September and previous October, the SPEI6 from previous September to current January, the SPEI9 from previous September to current April, and the SPEI12 from previous September to current July all had significantly positive effects on tree growth. Moreover, the Tmax of previous September had a negative effect on tree growth. The SPEI1 of current July was positively correlated with the chronology, albeit with a relatively low correlation coefficient (**Figure 4**).

### 3.4 Differences in the responses to two types of extreme climate events

**Figure 5:**
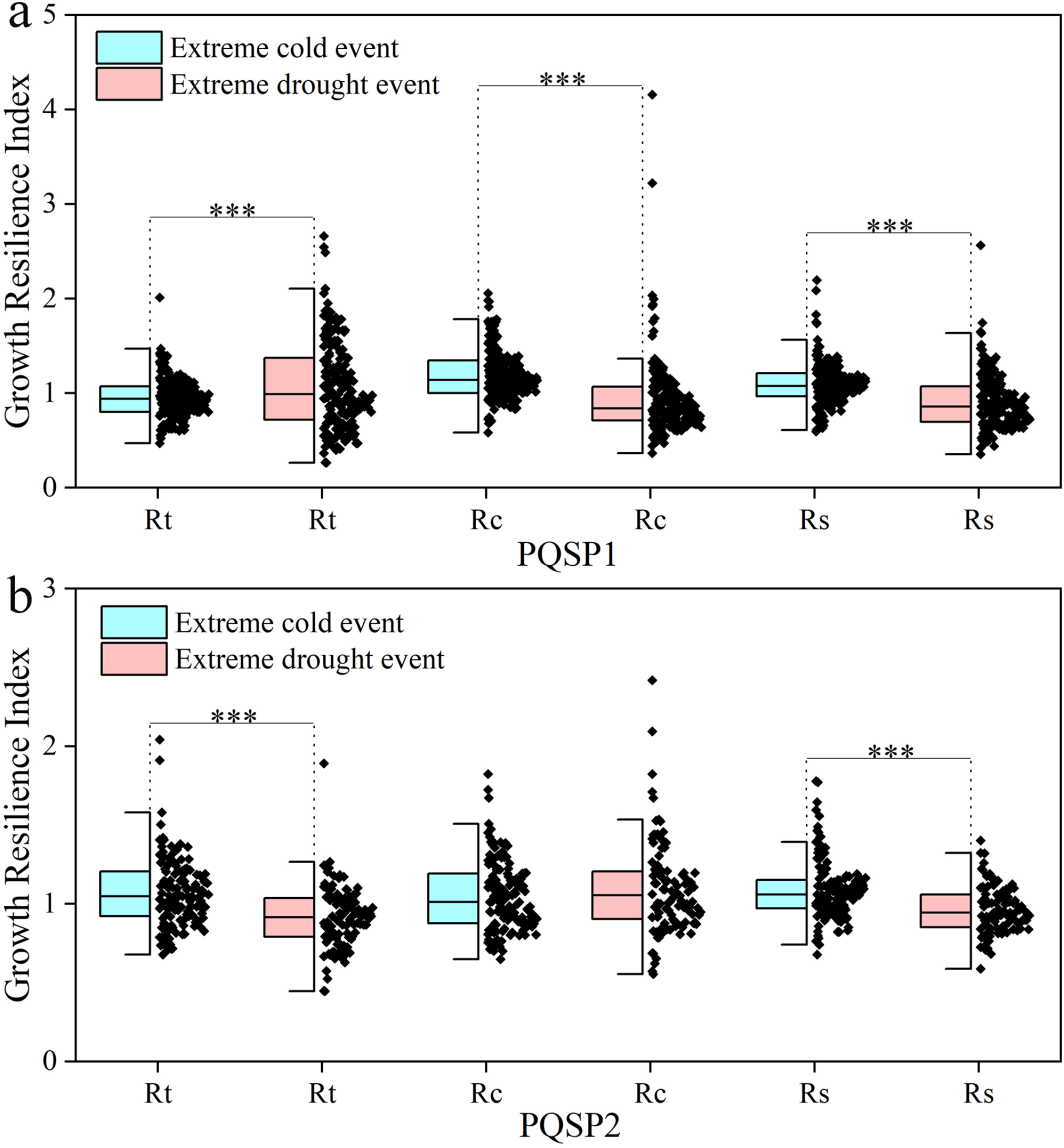
ANOVA results on the resilience of **(a)** PQSP1 and **(b)** PQSP2 to different types of extreme climate events. *** indicates a significant correlation at the 0.001 level.

We first compared the differences in resistance, recovery, and resilience of each stand to two types of extreme climate events. The results showed that for PQSP1, there were significant differences in growth resilience to both types of extreme climate events. Specifically, resistance to extreme cold was lower than that to extreme drought, whereas recovery and resilience to extreme cold were both higher than those to extreme drought. For PQSP2, both resistance and resilience to extreme cold were higher than those to extreme drought, while no difference was found in recovery (**Figure 5**).

**Figure 6:**
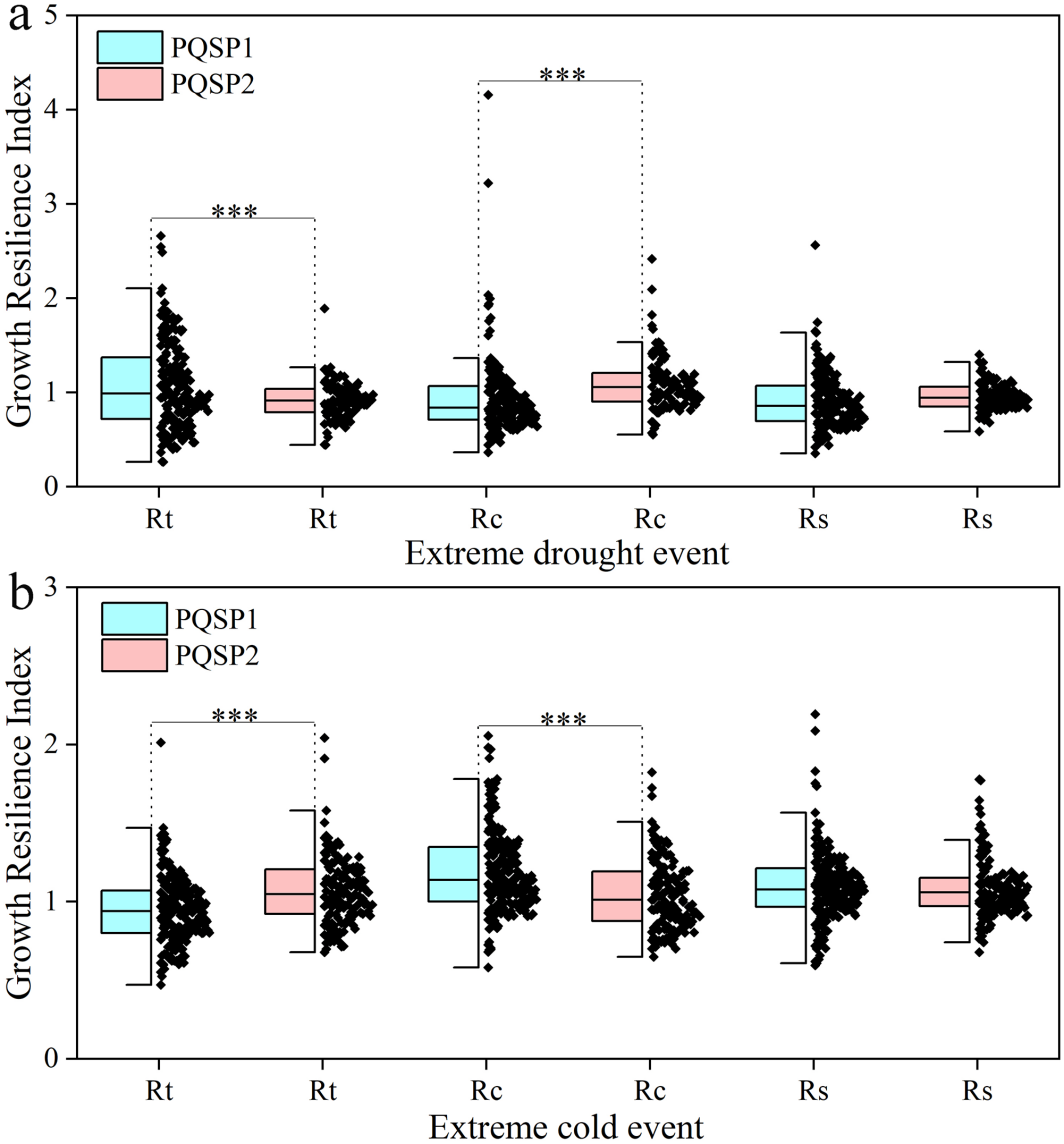
ANOVA results on differences in growth resilience to **(A)** extreme drought events and **(B)** extreme cold events between the two stands. *** indicates a significant correlation at the 0.001 level.

We also compared the differences in growth resilience to the same type of extreme event between different stand ages. For extreme drought events, the resistance of PQSP1 was higher than that of PQSP2, whereas the recovery showed the opposite pattern. No difference was found in growth resilience between the two stands. For extreme cold events, the resistance of PQSP1 was lower than that of PQSP2, whereas the recovery showed the opposite pattern. Similarly, there was no difference in growth resilience between the two stands (**Figure 6**).

## 4 Discussion

### 4.1 Growth-climate relationships across two stands

The two stands of different ages respond to climate in completely different ways. The radial growth of middle-aged stands exhibited a significant positive correlation with moisture conditions of growth season. This is primarily because middle-aged trees are in their most active growth phase, where cambial activity and cell expansion are highly sensitive to turgor pressure and water availability (Fritts, 2012; Leng et al., 2025; Zhang et al., 2023). Furthermore, middle-aged trees often possess shallower root systems compared to mature trees, making them more vulnerable to surface soil moisture deficits during the high-evapotranspiration period of mid-summer ( (Leng et al., 2025; Rubio-Cuadrado et al., 2024; Zang et al., 2014). This finding is consistent with previous studies on temperate forests, which reported that younger age cohorts maintain a higher sensitivity to growing-season precipitation as they lack the deep-water access of their older counterparts (Liu et al., 2023; Wiles et al., 2025; Zhang et al., 2023).

In contrast, the growth of mature stands is primarily governed by pre-growing season moisture levels. This highlights the superior buffering capacity of older trees. Mature trees possess well-developed root systems that can extract water from deeper soil layers, effectively bypassing immediate growing-season deficits that affect younger trees (Gao DX. et al., 2025; Zang et al., 2014). Furthermore, favorable moisture in the previous autumn allows for the accumulation of non-structural carbohydrate (NSC) reserves and optimal soil water recharge before dormancy (Anderegg et al., 2015; Jing et al., 2026), which in turn enable trees to enter the rapid growth period earlier in the following year. The negative correlation with previous September maximum temperature (Tmax) in mature trees further supports this: high temperatures at the end of the previous growth season likely increase maintenance respiration costs, depleting the carbon reserves necessary for initiating xylogenesis the following spring (Belokopytova et al., 2022; Li et al., 2024).

These Even negative correlation of October SPEI1 in middle-aged trees was observed, the positive influence of longer-term SPEI (9 and 12 months) in October indicates that hydrological recharge over the full year remains beneficial for subsequent growth. These divergent response patterns reflect different carbon allocation strategies (Trugman et al., 2018). These results also indicated that mature trees, with more developed storage tissues, rely heavily on these previous-year reserves to initiate early-spring xylogenesis, a process that is often less pronounced in younger trees (Li et al., 2024; Zhu et al., 2023).

### 4.2 Growth resilience to extreme cold and drought events

Our within-stand analysis reveals that middle-aged trees (PQSP1) are more vulnerable to immediate growth loss during cold events but possess superior recovery potential compared to their response to drought. This lower cold resistance likely stems from the earlier phenological timing (budburst) and higher tissue water content of younger trees, which exposes nascent organs to freezing during late spring frosts (Li et al., 2024; Vitasse et al., 2019). However, their superior recovery from cold reflects a difference in damage mechanisms. This disparity stems from the distinct damage mechanisms: extreme drought often causes xylem embolism and hydraulic failure, requiring several years of new wood formation to restore water transport (Duan et al., 2022; Rehschuh et al., 2020; Skelton et al., 2017). Conversely, extreme cold (such as late spring frost) primarily damages new foliage or buds (Príncipe et al., 2017; Vitasse et al., 2019). Middle-aged trees, characterized by high physiological vigor and phenological plasticity, can rapidly trigger a “secondary flush” or mobilize stored carbon to rebuild the canopy, allowing for a swift recovery within one to two years (Mediavilla and Escudero, 2009; Rubio-Cuadrado et al., 2025).

For mature stands (PQSP2), the higher resistance and resilience to cold compared to drought suggest that larger tree size acts as a thermal buffer (Zhu et al., 2015). Thicker bark and greater biomass provide insulation against extreme temperature drops, and older trees may benefit from a later budburst that avoids early-season frost (Vitasse et al., 2019). Notably, the lack of difference in recovery between the two stressors in mature trees suggests they are governed by a fixed biological limit (Esperon-Rodriguez et al., 2025). Regardless of the stress type, the high maintenance costs of a massive body and a slower metabolic rate constrain the ability of mature trees to exhibit the explosive post-disturbance growth surge seen in younger cohorts.

### 4.3 Age-related discrepancies and the resistance-recovery trade-off

Under extreme drought, the significant discrepancies in resistance and recovery between age groups illustrate a clear resistance-recovery trade-off. The higher resistance in middle-aged stand compared to mature stand may reflect more efficient stomatal regulation (isohydric behavior) in younger trees, preventing immediate cavitation (Hartmann et al., 2021). Mature trees, conversely, showed lower resistance but higher recovery, likely by leveraging their vast internal storage of water and NSC to re-establish physiological function once the drought terminated (Bouknine et al., 2026; Huang et al., 2023). The convergence of resilience suggests that while developmental stages follow different strategies: one emphasizing damage avoidance and the other emphasizing post-stress compensation, both cohorts maintain a comparable long-term capacity to return to pre-drought growth levels (Wang et al., 2022).

Under extreme cold, the trade-off is reversed. Middle-aged stand showed lower resistance due to phenological vulnerability but higher recovery driven by growth plasticity and meristematic vigor (Huang et al., 2021; Zhu et al., 2015). Mature trees leveraged their physical size for higher resistance but were slower to recover. The identical resilience across ages under cold stress further suggests that in this temperate population, the immediate growth loss and subsequent surges are balanced over time through size-dependent compensation mechanisms (DeSoto et al., 2020). This ensures the long-term structural persistence of the forest across all ontogenetic stages, even as individual trees utilize different survival strategies based on their age and size (Merlin et al., 2015).

## 5 Conclusion

This study provides robust evidence that stand age significantly modulates the climate-response pattern and growth resilience of *P. wilsonii*, with middle-aged and mature trees employing divergent physiological strategies to navigate climatic extremes on the Loess Plateau. Our findings reveal a fundamental resistance-recovery trade-off linked to developmental stages: middle-aged stands exhibit higher sensitivity to growing-season moisture and lower resistance to cold damage, yet they possess a superior capacity for post-cold recovery. In contrast, mature trees utilize a conservative stability strategy to bolster drought recovery and using their larger biomass as a thermal buffer to maintain higher cold resistance. While both age classes achieve comparable long-term resilience through these distinct strategies, their specific vulnerabilities necessitate differentiated management frameworks. Specifically, adaptive forestry should prioritize drought mitigation and density control for younger stands to alleviate moisture stress, while focusing on protecting the structural integrity and microclimatic buffering of mature forests to secure their function as stable carbon reservoirs in an increasingly uncertain climate.

## Data Availability

The raw data supporting the conclusions of this article will be made available by the authors, without undue reservation.

## References

[B1] Anderegg W. R. L. Schwalm C. Biondi F. Camarero J. J. Koch G. Litvak M. . (2015). Pervasive drought legacies in forest ecosystems and their implications for carbon cycle models. Science 349, 528–532. doi: 10.1126/science.aab1833 26228147

[B2] Beguería S. Vicente-Serrano S. M. (2017). “ SPEI: calculation of the Standardised Precipitation-Evapotranspiration Index (R package),” in R. Package Version 1 (7). Available online at: https://CRAN.R-project.org/package=SPEI (Accessed May 8, 2026).

[B3] Belokopytova L. V. Zhirnova D. F. Krutovsky K. V. Mapitov N. B. Vaganov E. A. Babushkina E. A. (2022). Species- and age-specific growth reactions to extreme droughts of the keystone tree species across forest-steppe and sub-taiga habitats of south Siberia. Forests 13, 1027. doi: 10.3390/f13071027 30654563

[B4] Bouknine A. Sarmoum M. Valeriano C. Hammou M. A. Mokhfi F. Tefiel H. . (2026). Resilient but declining: drought induced dieback of aleppo pine stands in western Algeria. J. For. Res. 37, 32. doi: 10.1007/s11676-025-01976-y 30311153

[B5] Bunn A. G. (2008). “ A dendrochronology program library in R (dplR),” in Dendrochronologia 26 (2), 115–124. doi: 10.1016/j.dendro.2008.01.002

[B6] Cai Q. Liu Y. Lei Y. Bao G. Sun B. (2014). Reconstruction of the March–August PDSI since 1703 AD based on tree rings of Chinese pine (Pinus tabulaeformis Carr.) in the Lingkong Mountain, southeast Chinese loess Plateau. Clim. Past 10, 509–521. doi: 10.5194/cp-10-509-2014

[B7] Chen S. Wang C. Ren Y. Zhang H. Zhou D. Li J. . (2023). Spatial autocorrelation patterns and influencing factors of tree radial growth in the secondary picea forest in guandi mountains, northern China. Acta Ecol. Sin. 43, 1572–1583. doi: 10.5846/stxb202203080550

[B8] DeSoto L. Cailleret M. Sterck F. Jansen S. Kramer K. Robert E. M. R. (2020). Low growth resilience to drought is related to future mortality risk in trees. Nat. Commun. 11, 1–9. doi: 10.1038/s41467-020-14300-5 31992718 PMC6987235

[B9] Ding H. Pretzsch H. Schütze G. Rötzer T. (2017). Size-dependence of tree growth response to drought for Norway spruce and European beech individuals in monospecific and mixed-species stands. Plant Biol. 19, 709–719. doi: 10.1111/plb.12596 28644576

[B10] Duan C.-Y. Li M.-Y. Fang L.-D. Cao Y. Wu D.-D. Liu H. . (2022). Greater hydraulic safety contributes to higher growth resilience to drought across seven pine species in a semi-arid environment. Tree Physiol. 42, 727–739. doi: 10.1093/treephys/tpab137 34718811

[B11] Esperon-Rodriguez M. Brookhouse M. Power S. A. Avi D. Baer T. Rymer P. D. . (2025). Urban tree growth and drought responses show evidence of climate resilience. Glob. Change Biol. 31 (6), e70281. doi: 10.1111/gcb.70281 PMC12146951 40485435

[B12] Fang O. Zhang Q. (2024). Stress triggers tree-growth rebound in global forests. Agric. For. Meteorol. 359, 110285. doi: 10.1016/j.agrformet.2024.110285 38826717

[B13] Fritts H. (2012). Tree Rings and Climate (Amsterdam: Elsevier).

[B14] Gao C. Yang B. Wang F. Li G. Ljungqvist F. C. Bräuning A. . (2025). Meta-analysis of climate effects on radial growth of Qinghai spruce in northwestern China. J. For. Res. 36, 92. doi: 10.1007/s11676-025-01884-1 30311153

[B15] Gao D.-X. Xaybouangeun N. Zaw Z. Yang R.-Q. Fan Z.-X. (2025). Growth resilience of pinus latteri to extreme drought events across aridity gradients in southern Laos. Glob. Ecol. Conserv. 57, e03424. doi: 10.1016/j.gecco.2025.e03424 38826717

[B16] Gao W. Liu J. Bao W. Duan F. He X. Gao D. . (2025). Extreme droughts decrease the growth and resilience of juniperus rigida in the northern edge but not in the southern. Agric. For. Meteorol. 362, 110387. doi: 10.1016/j.agrformet.2025.110387 38826717

[B17] Gou X. Zhang T. Yu S. Liu K. Zhang R. Shang H. . (2023). Climate response of picea schrenkiana based on tree-ring width and maximum density. Dendrochronologia 78, 126067. doi: 10.1016/j.dendro.2023.126067 38826717

[B18] Guan W. Xiong W. Wang Y. H. Yu P. T. He C. Q. Du A. P. . (2007). Stem diameter growth of Larix principis-rupprechtii and its response to meteorological factors in the north of Liupan Mountain. Scientia Silvae Sinicae 43, 1–6. doi: 10.1016/s1872-2032(07)60015-8

[B19] Han L. Camarero J. J. Jia G. Zhang Z. Chen L. (2025). Drought resilience and legacy effects in two forest tree species on loess plateau of China: growth and water-use efficiency under different drought conditions. For. Ecosyst. 13, 100297. doi: 10.1016/j.fecs.2025.100297 38826717

[B20] Hartmann H. Link R. M. Schuldt B. (2021). “ A whole-plant perspective of isohydry: stem-level support for leaf-level plant water regulation,” in Tree Physiol., vol. 41. , 901–905. doi: 10.1093/treephys/tpab011 33594416 PMC8827077

[B21] Huang J. G. Zhang Y. L. Wang M. H. Yu X. H. Deslauriers A. Fonti P. . (2023). A critical thermal transition driving spring phenology of Northern Hemisphere conifers. Glob. Change Biol. 29, 1606–1617. doi: 10.1111/gcb.16543 36451586

[B22] Huang X. Sun X. Jiang Y. Xue F. Cui M. Zhao S. . (2021). The radial growth of picea wilsonii was more restricted by precipitation due to climate warming on Mt. Guandi, China. Forests 12, 1602. doi: 10.3390/f12111602 30654563

[B23] Huang X. Wang L. Mo X. Jiang Y. (2024). Response to ENSO events in the radial growth of picea wilsonii in Guandi mountains, China. J. Ecol. Rural Environ. 40, 919–926. doi: 10.19741/j.issn.1673-4831.2023.0536

[B24] Italiano S. S. P. Camarero J. J. Borghetti M. Colangelo M. Rita A. Ripullone F. (2024). Drought legacies in mixed Mediterranean forests: analysing the effects of structural overshoot, functional traits and site factors. Sci. Total Environ. 927, 172166. doi: 10.1016/j.scitotenv.2024.172166 38575023

[B25] Jiang S. Zhao P. Ma Q. Liang H. (2025). Evidence of threat from short-timescale dry season drought on tree radial growth in China’s humid subtropical forest. Agric. For. Meteorol. 373, 110744. doi: 10.1016/j.agrformet.2025.110744 38826717

[B26] Jing M. Sun C. Liu Y. Wu X. Li Z. Wang Y. . (2026). Forest growth in central China: rapid recovery from short-term drought but persistent suppression after prolonged drought. For. Ecol. Manage. 610, 123675. doi: 10.1016/j.foreco.2026.123675 38826717

[B27] Leng Q.-N. Gong X.-W. Li M.-Y. Shi H. Li Z.-K. Hao G.-Y. (2025). Scale- and age-dependent climate response patterns revealed in pinus sylvestris var. mongolica and larix principis-rupprechtii plantations in northern China. For. Ecol. Manage. 595, 122973. doi: 10.1016/j.foreco.2025.122973 38826717

[B28] Li Z. Wang C. Gao G. Feng X. Lv Y. Wang X. . (2026). Climate warming induced pervasive growth decline in chinese pine populations of the loess plateau, China. Front. Plant Sci. 17-2026. doi: 10.3389/fpls.2026.1749887 41815426 PMC12971888

[B29] Li W. Zhu L. Zhu L. Jing M. Qian C. Zhu Y. . (2024). Old pinus massoniana forests benefit more from recent rapid warming in humid subtropical areas of central-southern China. J. For. Res. 35, 88. doi: 10.1007/s11676-024-01740-8 30311153

[B30] Liang H. Jiang S. Muhammad A. Kang J. Zhu H. Li X. . (2021). Radial growth response of picea crassifolia to climatic conditions in a dryland forest ecosystem in northwest China. Forests 12, 1382. doi: 10.3390/f12101382 30654563

[B31] Liang H. Li J. Ren Y. Wang L. Wu Z. (2026). Macroclimate determines whether tree growth at alpine treelines is primarily limited by temperature or precipitation in northern China. Dendrochronologia 95, 126455. doi: 10.1016/j.dendro.2025.126455 38826717

[B32] Liang R. Sun Y. Zhu Z. Li R. (2024). Tree characteristics, drought and microtopography modulate the response of subtropical cunninghamia lanceolata to drought. Eur. J. For. Res. 143, 1787–1804. doi: 10.1007/s10342-024-01728-3 30311153

[B33] Liu P. Hu S. Wei H. He W. Zhou Y. Wang Y. (2023). Response of radial growth of pinus sylvestris var. mongolica of different stand ages to climate and extreme drought events in the semi-arid region of western liaoning, northeast China. Front. For. Glob. Change 6, 1272477. doi: 10.3389/ffgc.2023.1272477

[B34] Liu Y. Zhang X. Song H. Cai Q. Li Q. Zhao B. . (2017). Tree-ring-width-based PDSI reconstruction for central inner Mongolia, China over the past 333 years. Clim. Dyn. 48, 867–879. doi: 10.1007/s00382-016-3115-6 30311153

[B35] Lloret F. Keeling E. G. Sala A. (2011). Components of tree resilience: effects of successive low‐growth episodes in old ponderosa pine forests. Oikos 120, 1909–1920. doi: 10.1111/j.1600-0706.2011.19372.x 40046247

[B36] Lu X. Geng Z. Zhu Z. Liang E. Lin W. Pandey J. . (2025). Growth resilience of juniper shrubs is higher after wet than after dry spells under contrasting climatic conditions. Reg. Environ. Change 25, 79. doi: 10.1007/s10113-025-02425-6 30311153

[B37] Mediavilla S. Escudero A. (2009). Ontogenetic changes in leaf phenology of two co-occurring mediterranean oaks differing in leaf life span. Ecol. Res. 24, 1083–1090. doi: 10.1007/s11284-009-0587-4 18171660

[B38] Merlin M. Perot T. Perret S. Korboulewsky N. Vallet P. (2015). Effects of stand composition and tree size on resistance and resilience to drought in sessile oak and Scots pine. For. Ecol. Manage. 339, 22–33. doi: 10.1016/j.foreco.2014.11.032

[B39] Piraino S. Molina J. A. Hadad M. A. Juñent F. A. R. (2022). Resilience capacity of araucaria araucana to extreme drought events. Dendrochronologia 75, 125996. doi: 10.1016/j.dendro.2022.125996 38826717

[B40] Popa A. Van Der Maaten-Theunissen M. Popa I. Badea O. Van Der Maaten E. (2024). Spruce suffers most from drought at low elevations in the carpathians, though shows high resilience. For. Ecol. Manage. 571, 122201. doi: 10.1016/j.foreco.2024.122201 38826717

[B41] Pretzsch H. Schuetze G. Uhl E. (2013). “ Resistance of European tree species to drought stress in mixed versus pure forests: evidence of stress release by inter-specific facilitation,” in Plant Biol., vol. 15. , 483–495. doi: 10.1111/j.1438-8677.2012.00670.x 23062025

[B42] Príncipe A. Van Der Maaten E. Van Der Maaten-Theunissen M. Struwe T. Wilmking M. Kreyling J. (2017). Low resistance but high resilience in growth of a major deciduous forest tree (fagus sylvatica L.) in response to late spring frost in southern Germany. Trees 31, 743–751. doi: 10.1007/s00468-016-1505-3 30311153

[B43] Rehschuh R. Cecilia A. Zuber M. Faragó T. Baumbach T. Hartmann H. . (2020). Drought-induced xylem embolism limits the recovery of leaf gas exchange in scots pine. Plant Physiol. 184, 852–864. doi: 10.1104/pp.20.00407 32820065 PMC7536670

[B44] Rubio-Cuadrado Á. Dorado-Liñán I. López R. Camarero J. (2025). A decade of lost growth in old trees: aging shapes the impacts of drought and late frost events on European beech. Agric. For. Meteorol. 370, 110601. doi: 10.1016/j.agrformet.2025.110601 38826717

[B45] Rubio-Cuadrado A. Montes F. Alberdi I. Canellas I. Aullo-Maestro I. Sanchez-Salguero R. . (2024). Analyses from stand to tree level allow disentangling the effects of age, size, origin and competition on tree growth sensitivity to climate in natural and afforested scots pine forests. Agric. For. Meteorol. 355, 110148. doi: 10.1016/j.agrformet.2024.110148 38826717

[B46] Serra-Maluquer X. Mencuccini M. Martínez-Vilalta J. (2018). Changes in tree resistance, recovery and resilience across three successive extreme droughts in the northeast iberian peninsula. Oecologia 187, 343–354. doi: 10.1007/s00442-018-4118-2 29589144

[B47] Skelton R. P. Brodribb T. J. McAdam S. A. M. Mitchell P. J. (2017). Gas exchange recovery following natural drought is rapid unless limited by loss of leaf hydraulic conductance: evidence from an evergreen woodland. New Phytol. 215, 1399–1412. doi: 10.1111/nph.14652 28620915

[B48] Sun B. Ma L. Liu T. Huang X. Zhou Y. (2021). Temperature reconstruction based on 361 year old dendrochronology of platycladus orientalis (L.) franco in the wula mountains, China. Quat. Int. 583, 94–102. doi: 10.1016/j.quaint.2020.12.026 38826717

[B49] Trugman A. T. Detto M. Bartlett M. K. Medvigy D. Anderegg W. R. L. Schwalm C. (2018). Tree carbon allocation explains forest drought-kill and recovery patterns. Ecol. Lett. 21, 1552–1560. doi: 10.1111/ele.13136 30125446

[B50] Vitasse Y. Bottero A. Cailleret M. Bigler C. Fonti P. Gessler A. . (2019). Contrasting resistance and resilience to extreme drought and late spring frost in five major european tree species. Glob. Change Biol. 25, 3781–3792. doi: 10.1111/gcb.14803 31436853

[B51] Wang B. Chen T. Li C. Xu G. Wu G. Liu G. (2022). Discrepancy in growth resilience to drought among different stand-aged forests declines going from a semi-humid region to an arid region. For. Ecol. Manage. 511, 120135. doi: 10.1016/j.foreco.2022.120135 38826717

[B52] Wiles G. Wiesenberg N. Pollock M. Denes C. Cooper T. Smith D. . (2025). Changing climate response of northeast ohio white oaks, USA: is it tree age or site age? Dendrochronologia 91, 126307. doi: 10.1016/j.dendro.2025.126307 38826717

[B53] Xie M. Cai Q. Liu Y. Ren M. Zhou Q. Zhang H. . (2024). Assessing climatic response and drought resilience in growth of pinus tabulaeformis carr. and tsuga chinensis pritz. on the southern slope of the qinling mountains. Glob. Ecol. Conserv. 53, e02999. doi: 10.1016/j.gecco.2024.e02999 38826717

[B54] Yan X. Zhang Z. Wu X. Huang M. (2025). Radial growth-climate correlations and resilience of robinia pseudoacacia plantations to drought on the chinese loess plateau. Front. For. Glob. Change 8-2025. doi: 10.3389/ffgc.2025.1608397

[B55] Zang C. Hartl-Meier C. Dittmar C. Rothe A. Menzel A. (2014). Patterns of drought tolerance in major european temperate forest trees: climatic drivers and levels of variability. Glob. Change Biol. 20, 3767–3779. doi: 10.1111/gcb.12637 24838398

[B56] Zhang J. Gou X. Rademacher T. Wang L. Li Y. Sun Q. . (2023). Interaction of age and elevation on xylogenesis in juniperus przewalskii in a cold and arid region. Agric. For. Meteorol. 337, 109480. doi: 10.1016/j.agrformet.2023.109480 38826717

[B57] Zhu L. Zhang J. Camarero J. J. Cooper D. J. Cherubini P. Yuan D. . (2023). Drivers and spatiotemporal patterns of post-drought growth resilience of four temperate broad-leaved trees. Agric. For. Meteorol. 342, 109741. doi: 10.1016/j.agrformet.2023.109741 38826717

[B58] Zhu L. Zhou T. Chen B. Peng S. (2015). How does tree age influence damage and recovery in forests impacted by freezing rain and snow? Sci. China Life Sci. 58, 472–479. doi: 10.1007/s11427-014-4722-2 25316045

